# Stochastic oscillators in biology: introduction to the special issue

**DOI:** 10.1007/s00422-022-00931-y

**Published:** 2022-04-26

**Authors:** J. MacLaurin, J. M. Fellous, P. J. Thomas, B. Lindner

**Affiliations:** 1 Department of Mathematical Sciences, New Jersey Institute of Technology, Martin Luther King Boulevard, Newark, New Jersey 07102 USA; 2 Department of Psychology and Biomedical Engineering, University of Arizona, 1503 E University, Tucson, Arizona 85721 USA; 3 Department of Mathematics, Applied Mathematics, and Statistics, Case Western Reserve University, 10900 Euclid Avenue, Cleveland, Ohio 44106 USA; 4 Department of Physics, Humboldt Universität zu Berlin, Newtonstr.15, 12489 Berlin, Germany

Rhythmicity and stochasticity are salient features of naturally occurring dynamical systems throughout biology and are particularly important in neuroscience. Examples in neuroscience range from the repeated firing of nerve cells driven by steady currents to the collective oscillations observed in the EEG; in physiology more broadly we see a mixture of regular and irregular rhythms in the beating of the heart, the action of the lungs, the vibrations of sensory hair bundles in the cochlea, and the movement of the limbs. On yet broader scales, ranging from the glycolytic oscillation in yeast to the circadian rhythm, the interplay of fluctuation and regularity structure many aspects of life.

The mathematical models that can describe stochastic oscillations are diverse and so is the role that noise plays in these systems. Important models include simple linear systems (quasi cycles, typically with a stable focus) endowed with an additive noise, limit-cycle systems that are perturbed by fluctuations, but also excitable or heteroclinic systems, in which oscillations are induced by noise. This means that the notion of *stochastic oscillators* relies on a variety of different mathematical expressions, one of the reasons why the observed phenomenology (noisy but somewhat periodic time series in biological observables) has time and again inspired theoreticians in the field. The papers of this special issue on stochastic oscillations are good examples of the diversity of approaches to the topic.

Stochastic oscillations may be characterized by more than one period. This is the case for models that show stochastic bursting, a phenomenon which is analytically investigated by Zheng and Pikovsky in the first contribution of this issue. Extending earlier results, the authors derive approximate expressions for auto- and cross-correlations in systems of noisy excitable units with multiple delayed feedback.

Delays play also a prominent role in the study by Powanwe and Longtin, who study bidirectional communication and phase relationships between two stochastic Wilson–Cowan models. The latter represent two local brain networks which affect each other with some temporal delay due to the finite transmission speed of action potentials. Powanwe and Longtin report on an interesting alternation in which one of the populations may lead with respect to phase and another with respect to the information flow - remarkably, the two roles do not have to coincide at a given time.

Networks of oscillators under the effect of fluctuations that are common to all units and of noise that is individual for each oscillator are studied by Aminzare and Srivastava. They put forward a number of surprisingly general analytical results for the conditions of synchronization in homogeneous networks (in which only common noise is present) and conditions for approximate synchronization for heterogeneous networks (for which also individual noise is taken into account) and illustrate their findings with numerical examples using coupled Van der Pol limit-cycle oscillators.

One important aspect of dynamical systems is how they respond to periodic and stochastic driving. In particular, what types of stochastic oscillatory responses emerge when subject to a variety of inputs (sinusoidal, Gaussian noise, square-wave, synaptic) as a current or conductance change? These questions are particularly pertinent for neurons that exhibit subthreshold oscillations and resonance because they are known to shape neural information transmission as well as the rhythms of neural networks. Pena and Rotstein present in this issue a thorough study of the responses of neuron models to different kinds of periodic and stochastic stimuli.

Winkler et al. provide a theoretical framework to calculate the phase response curve of distributed-delay-induced limit cycles with infinite-dimensional phase space. The technique is applied to the Wilson–Cowan oscillator model of excitatory and inhibitory neuronal populations under the two delay distributions and the phase response curves are calculated for both the Gaussian and log-normal delay distributions. The authors use the obtained phase response curves to derive phase interaction functions and determine the possible phase-locked states of multiple inter-coupled populations to illuminate different synchronization scenarios.

Zemlianova et al. present a biophysical model of how to keep count of a pacemaker in the presence of various forms of stochasticity using a system of bistable Wilson-Cowan units asymmetrically connected in a one-dimensional array. All units receive the same input pulses from a central clock but only one unit is active at any point in time. With each pulse from the clock, the position of the activated unit changes thereby encoding the total number of pulses emitted by the clock. This neural architecture maps the counting problem into the spatial domain, which in turn translates count to a time estimate. The authors then extend the model to a hierarchical structure to be able to robustly achieve higher counts.

In recent years numerous mathematical frameworks have been developed for understanding the interaction of stochastic and deterministic dynamics in rhythmic dynamical systems. Examples include generalization of the notion of isochrons based on surfaces of uniform mean first return time put forward by Schwabedal and Pikovsky and a notion of “asymptotic phase” for stochastic oscillators by Thomas and Lindner based on a spectral decomposition of the Koopman operator. The paper by Pérez-Cervera et al. in this issue makes a thorough comparison between the mean-return-time phase and the asymptotic phase of a stochastic oscillator. The authors develop a mathematical framework for determining both phases and the difference between them and illustrate them for three paradigmatic systems from computational neuroscience. The paper by Holzhausen et al. in this issue focuses solely on the mean-return-time phase and makes an interesting connection to the point processes that play such a prominent role in neuroscience. These authors investigate how the interval-correlation statistics change when spike times are counted by crossings of conventional thresholds (e.g., a voltage in an integrate-and-fire model) or by crossings of the mean-return-time isochrons and find that in the latter case the serial correlation coefficient of inter-crossing-event intervals must vanish, which is illustrated by a number of examples.

The papers of this special issue certainly do not exhaust the rich topic of stochastic oscillations in biology; however, we do hope that our selection of articles has filled a few gaps in knowledge, has shed some light on interesting mathematical phenomena, and will stimulate further theoretical and experimental studies of noisy oscillators.

